# Retinal Artery Occlusion Secondary to Buerger's Disease (Thromboangiitis Obliterans)

**DOI:** 10.1155/2017/3637207

**Published:** 2017-04-16

**Authors:** Erdem Eris, Mehmet Emin Sucu, Irfan Perente, Zeynep Alkın, Abdullah Ozkaya, Hatice Nur Tarakcioglu

**Affiliations:** Beyoglu Eye Training and Research Hospital, Istanbul, Turkey

## Abstract

*Purpose.* To report a case report of one patient suffering from retinal artery occlusion secondary to Buerger's disease, in order to raise awareness to this etiology in the differential diagnosis of retinal artery occlusion.* Methods.* A retrospective case report of a patient with retinal artery occlusion secondary to Buerger's disease. Data retrieved from the medical records included exposure, complaints, visual acuity, clinical findings and imaging, laboratory assessment, treatment, disease course, and visual outcome.* Results.* Diagnosis of retinal artery occlusion secondary to Buerger's disease was established based on ruling out other causes of retinal artery occlusion. Inflammatory retinal vascular disease, permanent vision loss, and macular atrophy were shown in this case.* Conclusion.* The very first case of central retinal artery occlusion (CRAO) in a 64-year-old male patient with Buerger's disease. Although diagnosing CRAO based on both fundoscopic and fluorescein angiographic findings is not difficult, revealing underlying condition of CRAO occasionally could be challenging.

## 1. Introduction

Although central retinal artery occlusion is rare, it is an important cause of vision loss in the elderly. The incidence is estimated to be 1 in 100,000 people and accounts for 1 in 10,000 ophthalmological outpatient visits [1]. The most likely etiology of a CRAO in general and to this case is a cholesterol embolus (Hollenhorst plaque). Hollenhorst plaques are typically refractile and orange and located at a bifurcation. They arise from ulcerated atheromas from the carotid arteries [2]. Patients with CRAO typically present with an acute, painless loss of vision, and 80% of affected patients have a final visual acuity of counting fingers or worse [3, 4]. Thromboangiitis obliterans (TAO) also known as Buerger's disease is a segmental occlusive inflammatory condition of arteries and veins, characterized by thrombosis and recanalization of the affected vessels [5]. It is a nonatherosclerotic inflammatory disease affecting small and medium sized arteries and veins of the upper and lower extremities [6]. In the literature, CRAO associated with Buerger's disease has not been shown in the literature. Awareness of CRAO's diagnosis was aimed by reporting this case.

## 2. Case Description

**Figure 1:**
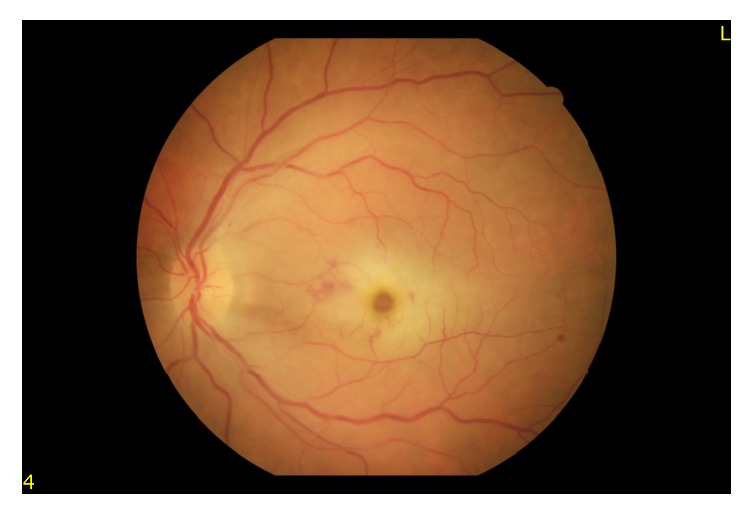
A cherryred spot in the macula in his left eye.

**Figure 2:**
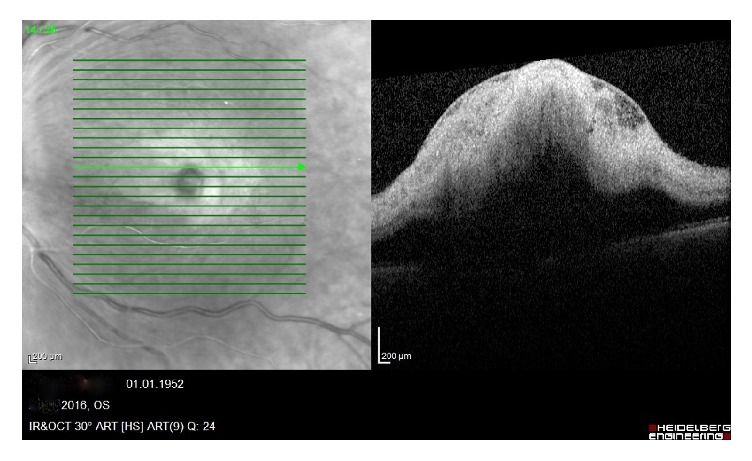
Increased reflectivity of the inner retinal layers in the left eye.

**Figure 3:**
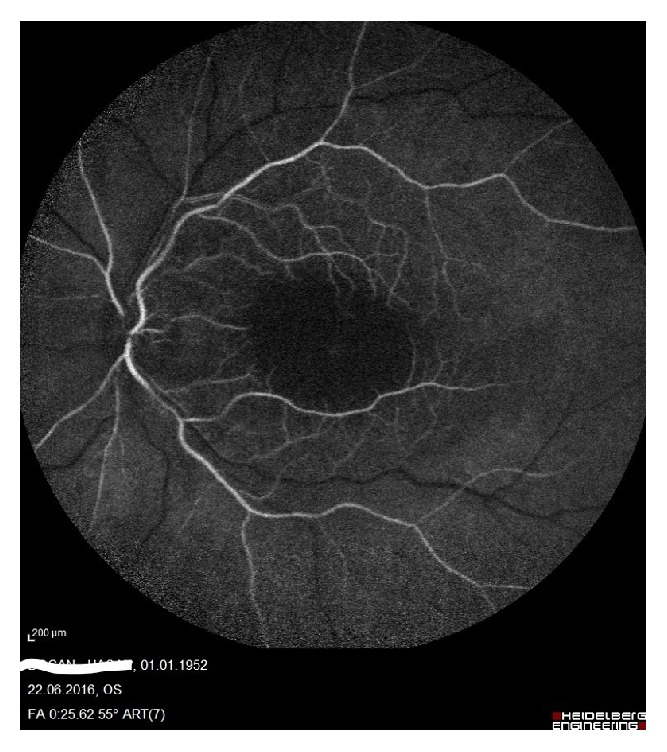
Early phase of fundus fluorescein angiography.

**Figure 4:**
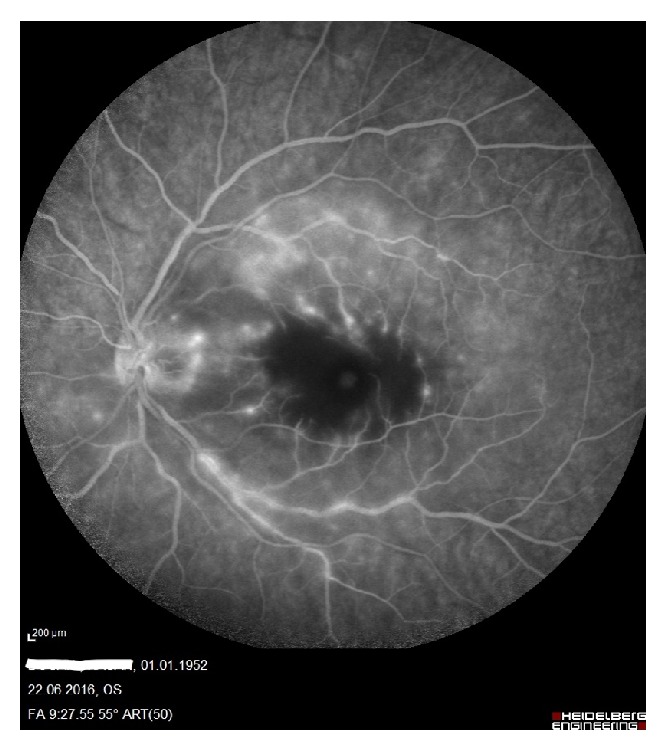
Late phase of fundus fluorescein angiography shows vascular hyperfluorescence.

**Figure 5:**
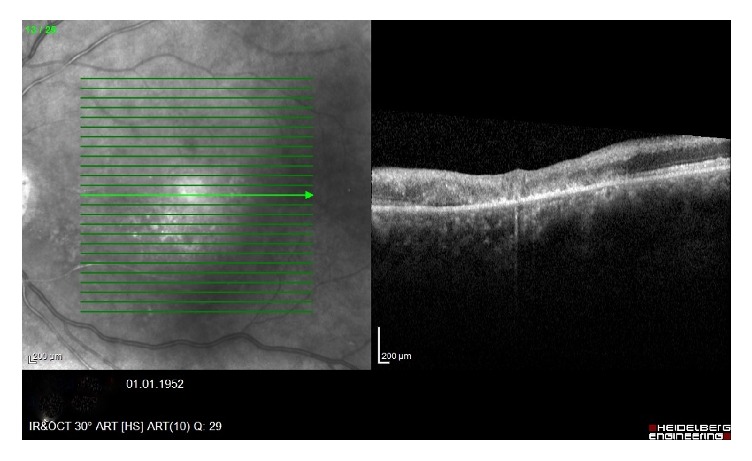
After 1 month of macular atrophy.

A 64-year-old man presented with painless, sudden vision loss of 5-day duration in his left eye. On the examination, visual acuity in his left eye was hand motion while in the right eye it was at 16/20. The anterior segment examination was normal and intraocular pressures were 15 mmHg in both eyes. The pupils were equal, with a relative afferent pupillary defect in the left eye. Dilated fundus examination showed an area of interpapillomacular retinal ischemia with whitish edges and a cherryred spot in the macula in his left eye (Figure 1). The right eye was normal. Optical coherence tomography (OCT) (Heidelberg Engineering, Inc., Vista, CA, USA) imaging showed increased reflectivity of the inner retinal layers in the left eye. Central macular thickness was 650 uM (Figure 2). Fundus fluorescein angiography (FFA) revealed nonperfusion in the macular area with late staining of small veins and arterioles and leakage (Figures 3 and 4). He had no history of systemic disease other than thromboangiitis obliterans (TAO) diagnosed for thirty-two years. He had left leg amputation caused by TAO twenty-five years ago. Also he had a history of smoking for forty-three years. Diagnostic workup including cardiovascular examination, electrocardiogram, echocardiogram, and Doppler evaluation of the neck vessels was within normal limits. Also hematological examination and other investigations related to CRAO were normal. On the basis of the diagnostic workup, laboratory evaluation, and patient history, occurrence of CRAO in the patient was considered due to TAO. Although limited benefit of hyperbaric oxygen therapy was considered, the patient was ordered hyperbaric oxygen therapy for 20 sessions. Following the treatment, his final visual acuity was 10/200 in the left eye and OCT imaging showed macular atrophy(Figure 5) at first month.

## 3. Discussion

Although the incidence of CRAO is estimated to be 1 in 100,000 people and accounts for 1 in 10,000 ophthalmological outpatient visits [1], this is the very first case of retinal artery occlusion with Buerger's disease for the following reasons:

(i) The most likely etiology of a CRAO in general and to this case is a cholesterol embolus (Hollenhorst plaque).

(ii) However, Buerger's disease is due to a recurring progressive inflammation and thrombosis of small and medium arteries and veins, and it is not an embolic disease.

In all patients over 50 years who have CRAO, it is essential to rule out temporal arteritis. And other etiologies were ruled out in the present case by fundoscopic examination and fluorescein angiographic, cardiovascular, and systemic findings. Although in this case an inflammatory process was seen on FFA like temporal arteritis (TA), TA was eliminated since patient's age and patient's sedimentation level were low. To the best of our knowledge if it was TA these parameters must be high. TAO is mainly found in 30–40-year-old males and cigarette smokers and is a clinical syndrome characterized by the development of segmental thrombotic occlusions and vasospasm of the medium and small vessels mostly in the lower extremities. Mesentery, coronary, and cerebral arteries could be rarely affected [7]. Bernardczykowa and Zawilski reported that changes in blood flow by vasospasm, arteriosclerosis, and thrombotic occlusions occur within the ocular arteries as well as systemic arteries in TAO [8]. By reporting a patient having CRAO caused by thromboangiitis obliterans we aimed to emphasize other etiologies of the retinal artery occlusion.
